# Parameters Affecting the Antimicrobial Properties of Cold Atmospheric Plasma Jet

**DOI:** 10.3390/jcm8111930

**Published:** 2019-11-09

**Authors:** Bih-Show Lou, Chih-Ho Lai, Teng-Ping Chu, Jang-Hsing Hsieh, Chun-Ming Chen, Yu-Ming Su, Chun-Wei Hou, Pang-Yun Chou, Jyh-Wei Lee

**Affiliations:** 1 Chemistry Division, Center for General Education, Chang Gung University, Taoyuan 33302, Taiwan; blou@mail.cgu.edu.tw (B.-S.L.); romeomonkey@msn.com (C.-W.H.); 2 Department of Nuclear Medicine and Molecular Imaging Center, Chang Gung Memorial Hospital, Taoyuan 33305, Taiwan; 3 Department of Microbiology & Immunology, Chang Gung University, Taoyuan 33302, Taiwan; chlai@mail.cgu.edu.tw; 4 Molecular Infectious Disease Research Center, Chang Gung Memorial Hospital, Taoyuan 33305, Taiwan; 5 School of Medicine, China Medical University and Hospital, Taichung 40402, Taiwan; 6 Department of Urology, University of Texas Southwestern Medical Center, Dallas, TX 75390, USA; 7 Center for Plasma and Thin Film Technologies, Ming Chi University of Technology, New Taipei 24301, Taiwan; bmw7458@gmail.com (T.-P.C.); jhhsieh@mail.mcut.edu.tw (J.-H.H.); cmchen@mail.mcut.edu.tw (C.-M.C.); ymsu73@gmail.com (Y.-M.S.); 8 Department of Materials Engineering, Ming Chi University of Technology, New Taipei 24301, Taiwan; 9 Plastic and Reconstructive Surgery, and Craniofacial Research Center, Chang Gung Memorial Hospital, Taoyuan 33305, Taiwan; chou.asapulu@gmail.com; 10 Department of Mechanical Engineering, Chang Gung University, Taoyuan 33302, Taiwan

**Keywords:** Taguchi method, antimicrobial efficiency, cold atmospheric-pressure plasma jet (CAPJ), *Escherichia coli*, DNA double-strand breaks, scanning electron microscopy

## Abstract

Using the Taguchi method to narrow experimental parameters, the antimicrobial efficiency of a cold atmospheric plasma jet (CAPJ) treatment was investigated. An L9 array with four parameters of CAPJ treatments, including the application voltage, CAPJ-sample distance, argon (Ar) gas flow rate, and CAPJ treatment time, were applied to examine the antimicrobial activity against *Escherichia coli* (*E. coli*). CAPJ treatment time was found to be the most influential parameter in its antimicrobial ability by evaluation of signal to noise ratios and analysis of variance. 100% bactericidal activity was achieved under the optimal bactericidal activity parameters including the application voltage of 8.5 kV, CAPJ-sample distance of 10 mm, Ar gas flow rate of 500 sccm, and CAPJ treatment time of 300 s, which confirms the efficacy of the Taguchi method in this design. In terms of the mechanism of CAPJ’s antimicrobial ability, the intensity of hydroxyl radical produced by CAPJ positively correlated to its antimicrobial efficiency. The CAPJ antimicrobial efficiency was further evaluated by both DNA double-strand breaks analysis and scanning electron microscopy examination of CAPJ treated bacteria. CAPJ destroyed the cell wall of *E. coli* and further damaged its DNA structure, thus leading to successful killing of bacteria. This study suggests that optimal conditions of CPAJ can provide effective antimicrobial activity and may be grounds for a novel approach for eradicating bacterial infections.

## 1. Introduction

Cold, or non-thermal, atmospheric-pressure plasma jets (CAPJ) have gained attention in biomedical applications [1,2,3,4,5,6] due to unique characteristics, comprising of a complex plasma chemistry without the need for elevated gas temperatures as required for traditional thermal plasma [7,8]. In addition, the high energy electrons of CAPJ can produce reactive oxygen species (ROS) with slightly higher temperature than ambient environment in an open air. The ROS produced by CAPJ plays a significant role in promising inactivation of bacteria [9,10], which can be used in developing antimicrobial treatments for infectious diseases or in sterilization of reusable thermal sensitive medical devices to prevent the outbreak of antibiotic-resistant bacteria [11]. Although these properties have led to extensive use of CAPJ in material processing and biomedical applications, consistent use of CAPJ without risks remains difficult. As the performance of CAPJ is dependent on the operational parameters, understanding how each parameter impacts the plasma’s properties is necessary and can allow for adjustment of the plasma for use in different applications.

*E. coli* is often used as an indicator of hygiene and safety in food products [12], and it is commonly found in community and hospital-acquired infections. *E. coli* is one of the most common and deadly pathogens causing pediatric urinary tract infection, intra-abdominal infection, or acute lung injury [13,14,15,16] and can lead to life-threatening intestinal infections in immunocompromised patients [17]. In addition, empiric antibiotic therapy may be ineffective with multidrug-resistant *E. coli* which will delay care and may cause further harm to the patient [18,19]. Therefore, we selected *E. coli* to investigate the antimicrobial efficiency of CAPJ treatment. The aim of this work is to develop the interventions by CAPJ treatment to prevent *E. coli*-induced diseases.

The influence of operational parameters of CAPJ, such as operation power, CAPJ-sample distance, gas mixtures composition, and treatment periods need to be determined to generate consistent results when using CAPJ. In general, a large number of experiments are required to optimize the processing parameters to improve the antimicrobial efficiency by changing one parameter value at a time. Such approach is time consuming, costly, and labor intensive. In this study, a systematic and efficient approach using the Taguchi experimental method design [20,21,22,23] is applied to the CAPJ system in determining the optimal process parameters for the highest antimicrobial efficiency. The optimal condition of CAPJ was determined experimentally and the antimicrobial mechanism of CAPJ was explored using DNA damage assay and microbial microstructure analysis.

## 2. Experimental Section

### 2.1. Plasma Experimental Setup

**Figure 1:**
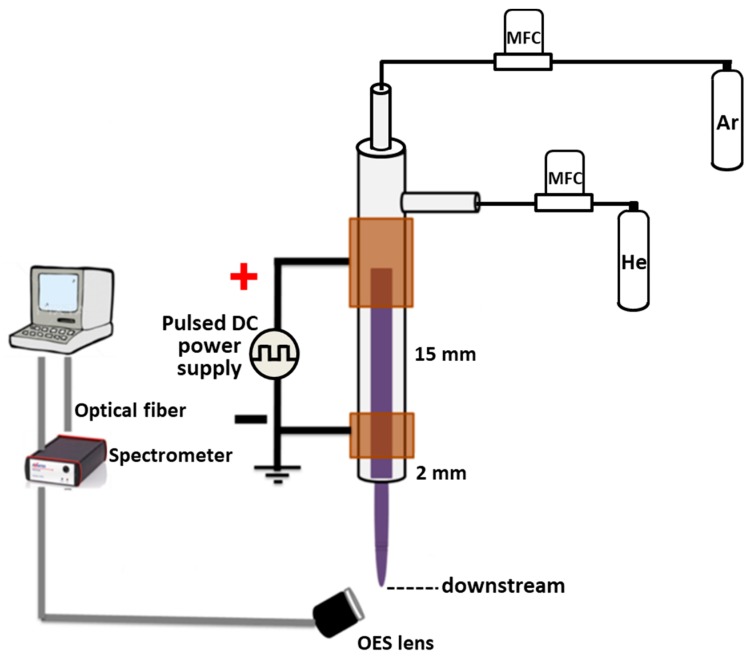
The setup of a cold atmospheric plasma jet (CAPJ).

Figure 1 depicts the setup of the CAPJ used. A quartz tube with 4 mm inner diameter was covered with two parallel cylindrical pure copper electrodes, which were connected with a high voltage pulsed direct current (DC) power supply for generating the plasma jet [24]. The distance between two Cu electrode was 15 mm. The frequency and the duty cycle of the power supply were fixed at 10,000 Hz and 50%, respectively. In this work, the He gas flow rate was kept at 5 slm (standard liter per min), and the argon gas flow rate was changed from 0 to 500 sccm (standard cubic centimeter per min). Taguchi method with L9 experiment was applied to discover the optimal sterilization effect. The CAPJs were generated under four different processing parameters in three various conditions as listed in Table 1, including the applying voltage of 6.5, 7.5, and 8.5 kV; the distance between CAPJ downstream and sample surface of 10, 20, and 30 mm; the CAPJ treating time of 60, 180, and 300 s; and the Ar gas flow rates of 0, 200, and 500 sccm. The radical compositions at the CAPJ downstream of each experiment was detected by an optical emission spectrometer (OES, AvaSpec ULS2048L, Avantes, Louisville, CO, USA) in the wavelength ranging from 200 to 1100 nm. The integration time for the OES detection was 80 ms.

### 2.2. Bacterial Strain and Culture

*E. coli* (ATCC 25922^TM^) was used as a reference strain, which was described elsewhere [25]. The bacteria were routinely cultured in Luria–Bertani (LB) broth (Becton Dickinson, Franklin Lakes, NJ, USA) at 37 °C for 24 h to reach the logarithmic phase and performed in the following experiments.

### 2.3. In Vitro Growth Inhibition Assay

*E. coli* was cultured in LB broth at 37 °C for 24 h. The bacterial suspension was adjusted to an optical density of 1.0 at 600 nm (OD_600_), which corresponded to 1 × 10^5^ colony-forming units (CFUs)/mL. The bacteria were untreated by CAPJ under various combinations of experimental parameters assigned as S1–S9 revealed in Table 2. The untreated *E. coli* was assigned as S0 (control). The bacteria were serial diluted in phosphate buffer saline (PBS) and plated onto LB agar plates. After incubation at 37 °C for 24 h, the viable CFUs were counted. The bactericidal activity was represented as a percentage of CAPJ treated one divided by the untreated control group (S0). The results were expressed as the means of three independent experiments performed in duplicate.

The bactericidal activity = (N_x_/N_0_) × 100%

where N_0_ and N_x_ are the number of the viable bacteria on an untreated control S0 and CAPJ treated S_x_ (x = 1–9) experiment after incubation at 37 °C for 24 h, respectively.

### 2.4. Taguchi Method

Four control factors of the Taguchi experiment including (A) application voltage (kV), (B) CAPJ downstream-sample distance (mm), (C) Ar gas flow rate (sccm), and (D) CAPJ treatment time (s) were applied to analyze the influence of the sterilization parameters on *E coli.* Three levels were considered for each factor and shown in Table 1. Table 2 represented the Taguchi design and structure of L9 orthogonal array reflecting the parameters and levels in Table 1. The higher the better concept was applied to estimate the significant parameters of CAPJ sterilization treatment by data analysis and signal-to-noise (S/N) ratios. The contribution percentages of individual parameters were determined by analysis of variance (ANOVA) [26]. The S/N ratio is defined as follows:

$$\frac{S}{N}=-10\times log\left[\frac{1}{n}\overset{n}{\underset{i=1}{\sum }}\frac{1}{y^{2}}\right]$$

where *n* is the number of experiments and *y* is the observed data.

A confirmation test was carried out to validate the Taguchi’s optimization approach. The summary statistic S/N at optimal conditions was calculated after the sanative parameters, which can be used to determine the optimal condition.

(S/N)_opt_ = m + (m_x_ − m) + (m_y_ − m)

where m is the overall mean, and m_x_ and m_y_ are the mean effect sensitive parameters at the optimal level [19]. The L9 orthogonal array for sample designation and detailed CAPJ treated parameters was tabulated in Table 3, which was the designed L9 orthogonal array in Table 2 filled with the corresponding parameters assigned in Table 1.

### 2.5. Bacterial Viability Assay

The LIVE/DEAD Bacterial Viability Kit (Thermo Fisher Scientific, Camarillo, CA, USA) was subjected to analyze the viability of bacterial populations based on the membrane integrity [27]. Two nucleic acid fluorescent dyes, SYTO9 and propidium iodide (PI), were used to determine the bacterial viability. The *E. coli* bacteria suspensions, which were untreated (S0) and CAPJ treated under S10, were collected and washed with PBS. The prepared samples were stained with SYTO9 and PI, according to the manufacturer’s instructions. The stained bacteria were then analyzed by a confocal laser scanning microscope (LSM 780; Carl Zeiss, Göttingen, Germany). The quantification of fluorescence intensity was performed by using a FACSCalibur flow cytometer (Becton Dickinson, San Jose, CA, USA).

### 2.6. DNA Damage Assay

A plasmid DNA, pGL3 (2 μg/μL) (Promega, Madison, WI, USA) was untreated (S0) or S10 CAPJ treated. Both the S0 and S10 CAPJ treated plasmids were then dissolved in DNA suspension buffer. DNA solution was loaded on 1.0% agarose gel for electrophoresis. Ethidium bromide-stained DNA was visualized under UV light. Photograph was taken by using a UV transilluminator (Azure Biosystems c400; Dublin, CA, USA).

### 2.7. Field Emission Scanning Electron Microscope Analysis

The morphologies of untreated S0 and S10 CAPJ treated bacteria were analyzed using a field-emission scanning electron microscope (FE-SEM, JSM 6701F, JEOL, Akishima, Japan). The bacteria samples were fixed in slides with 2% glutaraldehyde for 2 h, followed by washing with saline solution, and then exposed to 25%, 50%, and 75% of ethanol for 20 min, respectively, and finally immersed in 100% of ethanol for one hour. The slides were dried using a critical point dryer for 2 h afterwards. The bacteria samples were coated with a thin platinum layer around 5 nm thick by a sputter system (JFC 1600, JEOL, Akishima, Japan).

### 2.8. In Vivo Evaluation

Animal experiments were performed in accordance with the ethical standard approved by the Institutional Animal Care and Use Committee, Chang Gung University (Approval No. CGU105-032). Three male Sprague-Dawley (SD) rats with body weight range of 550 ± 30 g were study. The animals were housed under controlled conditions of temperature of 21–22 °C, relative humidity of 55% to 65%, and a 12 h light/dark cycle with artificial lighting. The animals received a standard feed and water ad libitum and were acclimatized under the aforementioned conditions before wounding experiment to create one full-thickness wounds with 17 mm in diameter on each side of rat’s shoulder as described in literature [28,29]. The right-side wounds were treated with S10 CAPJ, and the left side wounds were kept untreated as control (S0). Two bacterial swabs were immediately taken from each superficial wound site right after CAPJ treatment on day 0 and day 4. Swabs were immediately immersed in 3 mL PBS and inoculated 200 μL sample on LB agar plates. After incubation at 37 °C for 24 h, the CFUs were counted.

### 2.9. Statistical Analysis

During the ANOVA calculation, the relation of between-group comparisons was performed using the chi-square with Fisher exact test by SPSS program (version 18.0, SPSS, Inc., Chicago, IL, USA). A *p*-value less than 0.05 was considered statistically significant.

## 3. Results

### 3.1. Plasma Characterization

**Figure 2:**
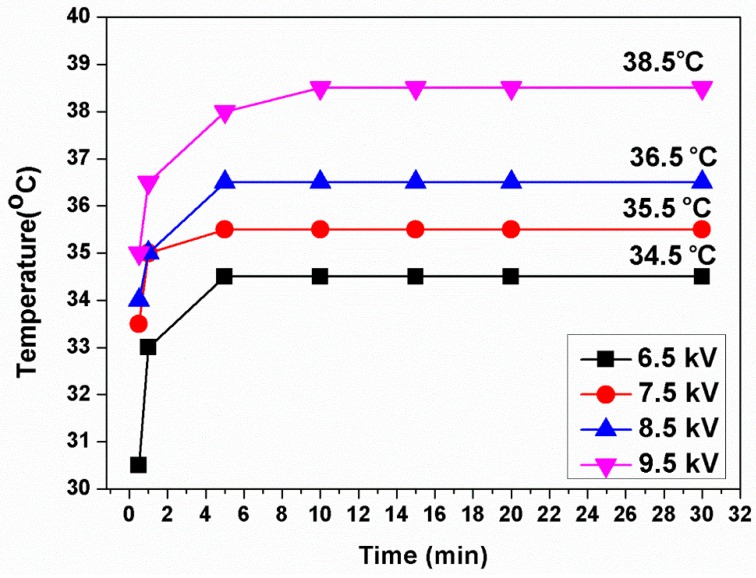
CAPJ temperature keeps steady for at least 30 min. Temperature changes of CAPJs operated at different applied voltages ranging from 6.5 to 9.5 kV under a fixed He gas flow rate of 5 slm. The temperature was detected at the position of 1 cm below the downstream of CAPJ.

The temperature of He-based CAPJ was measured at the distance of 10 mm from the nozzle, and it rose continually for the first 5 min and then kept constant for at least 30 min under pure He gas flow rate of 5 slm, as shown in Figure 2. The temperatures increased from 34.5, 35.5, 36.5 to 38.5 °C when the applying voltages were increased from 6.5, 7.5, 8.5 to 9.5 kHz, respectively.

**Figure 3:**
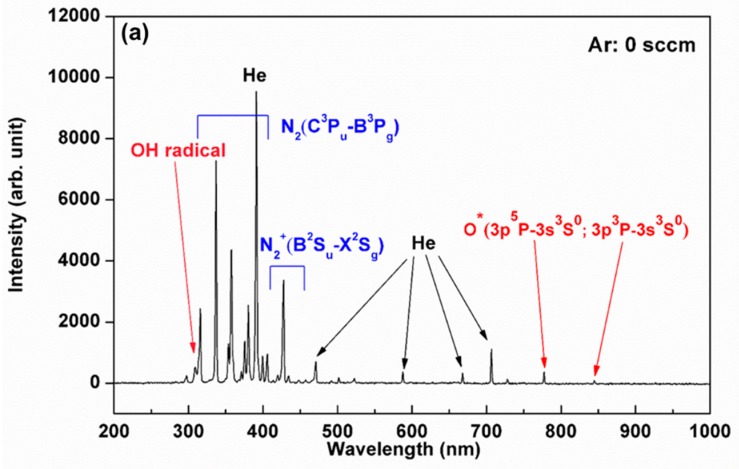

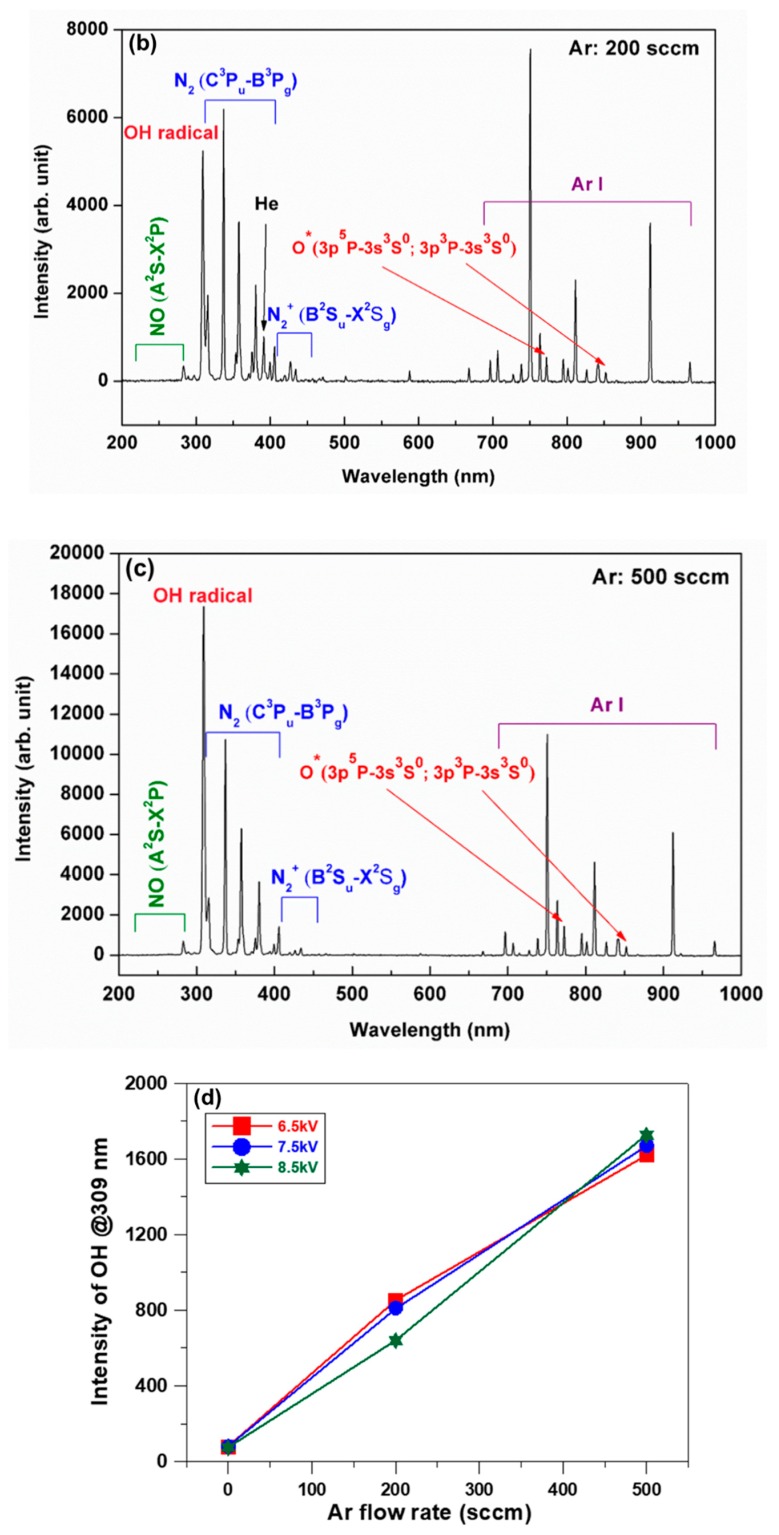
CAPJ plasma characterizes by OES. The OES spectra of the CAPJs operated at a fixed applied voltage of 6.5 kV and fixed He gas flow rate of 5 slm and (**a**) without Ar gas inlet, (**b**) with 200 sccm Ar gas and (**c**) with 500 sccm Ar gas, and (**d**) the dependence of Ar flow rate on the intensity of OH radicals @ 309 nm.

The compositions of ROS generated by CAPJ are mainly dependent on both the working gas and the atmospheric air. The compositions, therefore, can be controlled by the working gas mixture. Figure 3a–c illustrate the excited and radiant species in optical emission spectroscopy (OES) spectra under the mixtures of various Ar gas flow rates of 0, 200, and 500 sccm into a fix He gas flow rate of 5 slm with the application voltage of 6.5 kV. The emission lines between 300 and 400 nm, 450 and 700 nm and 700 and 900 nm were dominated by the nitrogen (N), He, and Ar atoms, respectively. Both hydroxyl radical (OH) emission at 309 nm and nitrogen monoxide (NO) emission at 283 nm are of particular interest because they might play an effective role for bacterial growth inhibition [1,30,31]. The intensities of OH radicals remarkably increased with increasing Ar gas flow rate but were unaffected at various application voltages, as observed in Figure 3d. It is also important to point out that the temperature of each Ar added CAPJ test was below 38.5 °C.

### 3.2. CAPJ Possess Bactericidal Activity

**Figure 4:**
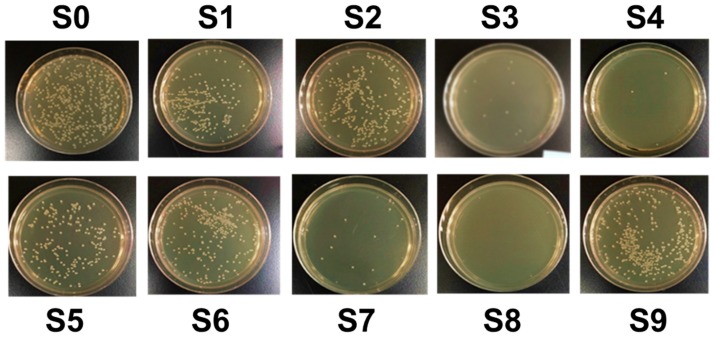
Bacterial colonies evaluate the bactericidal activity of CAPJ operating parameters. Photos of bacterial colonies of control S0 and CAPJ treated samples (**S1**) to (**S9**) with a combination of different Taguchi experimental parameters. The visualized colonies grew on the LB agar plates and were counted and represented in CFU as tabulated in Table 4.

The bactericidal activity was investigated under various plasma parameters designed using the Taguchi method shown in Figure 4 and summarized in Table 4. Comparing with the untreated control sample S0, the bactericidal activities against *E coli* by CAPJ treated under S1 to S9 were 45.7%, 31.3%, 90.6%, 92.8%, 53.2%, 37.7%, 85.9%, 100%, and 22.6%, respectively.

### 3.3. Analysis of the Bactericidal Activity by Using Taguchi Method

**Figure 5:**
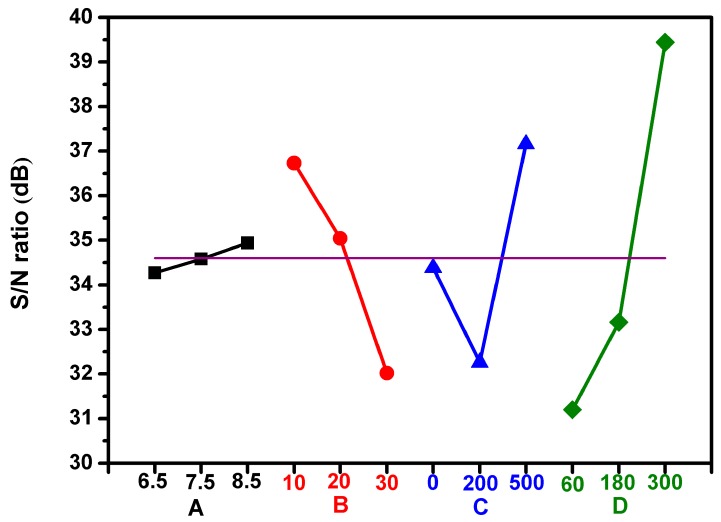
Taguchi analysis determines the best set of parameter combination. The effect diagrams for antimicrobial of CAPJ are based on the higher S/N and the better efficiency. Four factors include A: applying voltage (kV), B: CAPJ-sample distance (mm), C: Ar gas flow rate (sccm), and D: CAPJ treated time.

The four important operating parameters of CAPJ to achieve desired performance considered in this study were application voltages, CAPJ-sample distance, Ar gas flow rate, and CAPJ treatment time, which are noted as A, B, C, and D, respectively, in Table 1, and each was given three levels. The greater S/N value corresponded to the better performance regardless of the category of the performance characteristics [26]. Therefore, parameters with high S/N value and the better efficiency were selected to define the optimal level of the operating parameters. The average S/N ratios for each level of each parameter, in terms of bactericidal activity, were shown in Figure 5. The horizontal red line was the overall mean of the S/N values. The best combination of process parameters corresponding to the bactericidal activity was found to be A3B2C3D3, which correlates to application voltage of 8.5 kV, CAPJ-sample distance of 10 mm, Ar gas flow rate of 500 sccm, and CAPJ treatment time of 300 s. Furthermore, the CAPJ treatment time was discovered as the most impactful factor for bactericidal activity because of the greatest range of outcomes between the three experimental levels.

The relative significance of each parameter was investigated by ANOVA to estimate their contributions. The ANOVA results of the control factors, A, B, C, and D were calculated for the bactericidal activity and the degree of freedom, sum of squares, variance, and percentage contributions are shown in Table 5. Higher percentage contribution correlated to more significant influence of the overall process. Factor D, the CAPJ treatment time, was the most impactful with the highest variance of 4.70 and a percentage contribution of 76.5%. This was followed by factor C, Ar gas flow rate, and factor B, CAPJ-sample distance, with contributions of 12.1% and 11.2%, respectively. Meanwhile, the contributions of 0.2% for factor A, application voltage, is obtained implying its insignificant role.

### 3.4. Validation of Bactericidal Activity Conferred by CAPJ Treated under S10

**Figure 6:**
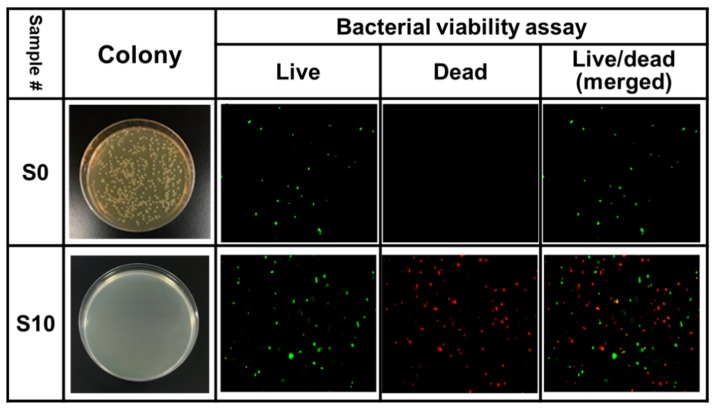
S10 CAPJ induces bacterial death. Photos of bacterial colonies of control S0 and confirmation test of CAPJ treated under S10, fluorescence live/dead bacterial viability assay images of *E. coli* without CAPJ (S0) and with CAPJ treated under S10.

Based on the optimal condition deduced from the Taguchi method, the CAPJ condition with the application voltage of 8.5 kV, CAPJ-sample distance of 10 mm, He/Ar gas flow of 5 slm/500 sccm, and CAPJ treated time of 300 s was assigned as S10, and a confirmation test was performed to validate the conclusions on the previous discovery. As compared with the control S0, the bactericidal activity of S10 was up to 100% in Figure 6.

To further verify whether the S10 CAPJ possesses potent bactericidal activity, a LIVE/DEAD Bacterial Viability assay was employed. As shown in Figure 6, dead bacteria, appearing with red fluorescence, were rarely observed in the control group, but those bacteria treated with S10 CAPJ had significantly more dead bacteria presented in the lower panel of Figure 6. These results confirmed that the S10 CAPJ had the most effective bactericidal activity in the treatment of bacterial pathogens and indicated that the Taguchi method is handy to find the optimal parameters on the bactericidal activity by CAPJ.

### 3.5. CAPJ Treatment Induced DNA Double-Strand Breaks (DSB) and Disruption of Cell Wall Integrity

**Figure 7:**
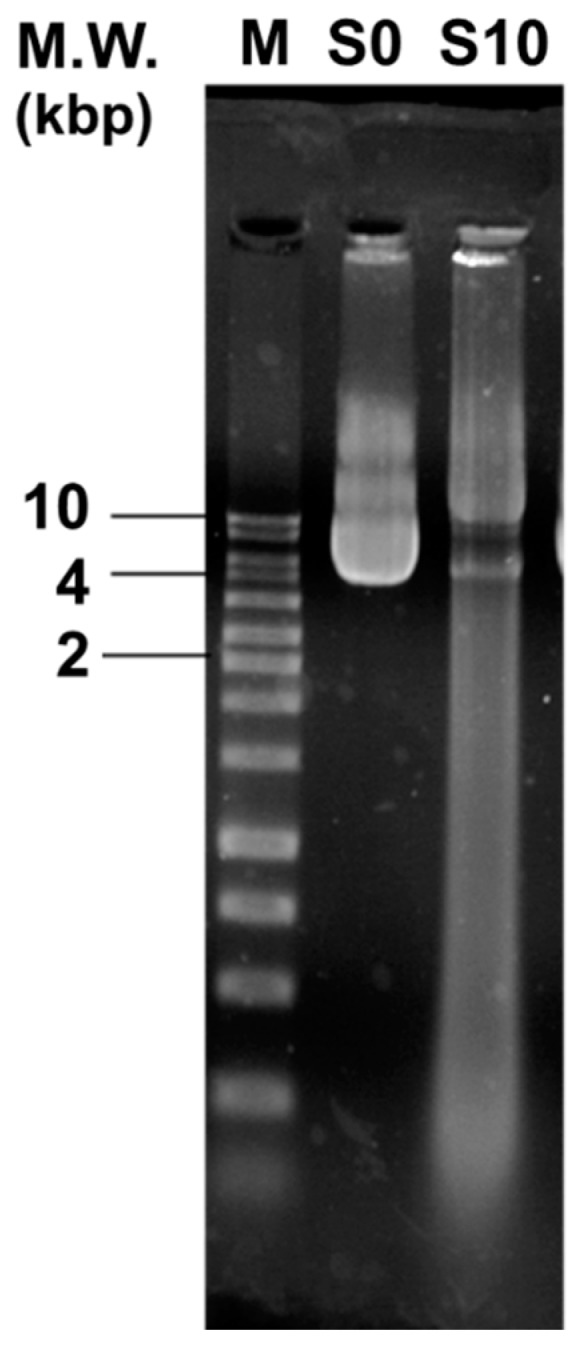
S10 CAPJ induces DNA double-strand breaks (DSB). The pGL3 (2 μg/μL) was untreated (S0) or treated with S10 CAPJ. The plasmid DNA was loaded on 1.0% agarose gel for electrophoresis. Ethidium bromide-stained DNA was visualized under UV light. The positions of the size markers are shown at left of the image. M, DNA marker.

**Figure 8:**
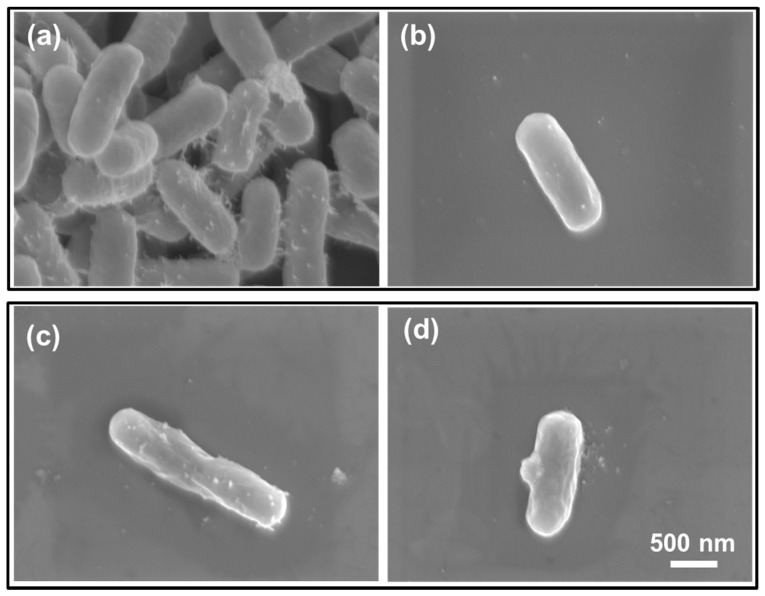
S10 CAPJ disrupts bacterial cell wall integrity. The FE-SEM images of the bacterial morphologies before CAPJ treatment (**a**,**b**) and after S10 CAPJ treatment (**c**,**d**).

To further explore the mechanism of the bactericidal activity by CAPJ treatment, we conducted a DNA electrophoresis assay. As shown in Figure 7, the untreated plasmid DNA (S0) was intact and exhibited the supercoiled and circular forms. In contrast, clear smear DNA fragments were shown in the plasmid DNA treated with S10 CAPJ, indicating that CAPJ contributed to induce DSB in bacteria. To strengthen our findings, the morphologies of *E. coli* were visualized by FE-SEM after bacteria was treated by S10 CAPJ. In the control group without CAPJ treatment, the bacterial shape and cell wall showed intact morphologies (Figure 8a,b). By contrast, FE-SEM images of the bacteria treated with S10 CAPJ exhibited a shriveled and burst appearance on the bacterial surface (Figure 8c,d). Taken together, these results demonstrated that CAPJ treated under S10 sustainably inhibited bacterial growth by inducing DSB and disrupting cell wall integrity.

### 3.6. In Vivo Evaluation

**Figure 9:**
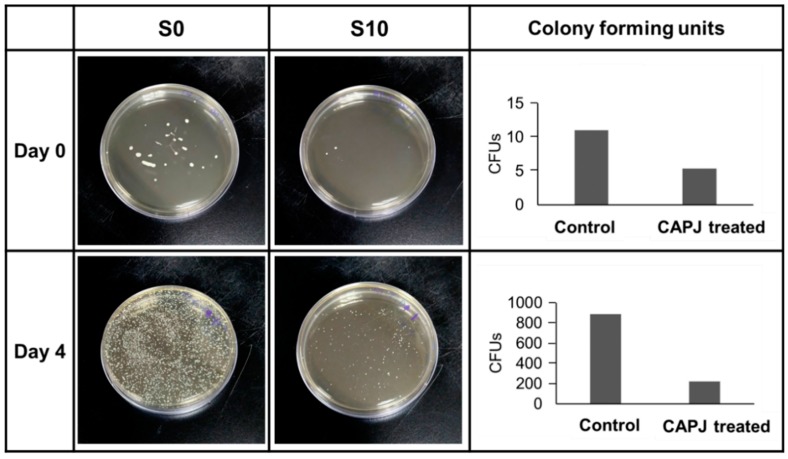
S10 CAPJ reduces bacterial infection in the wound. Rat wound was untreated or treated with S10 CAPJ, and the bacterial loads in the wound were counted on days 0 and 4. Viable bacteria were represented as colony forming units (CFUs).

Bactericidal activity in vivo evaluation by CAPJ treatment was presented in Figure 9. SD rates were used to create wound exposure and analyze the bacterial infection as described in the Experimental Section. The wounds were either untreated (S0) or treated with S10 CAPJ, and wound exudates were collected on days 0 and 4. Our results showed that S10 CAPJ treatment remarkably decreased bacterial load on day 0 when compared to S0. Most importantly, this effect is still seen on day 4, which showed that the bacterial infection continued to be reduced in rat treated with S10 CAPJ.

## 4. Discussion

Infectious disease remains a great challenge in the field of public health as it is among the top 10 causes of death and also leads the cause of disability [32]. Inadequate antibiotic therapy worsens the control of pathogenic microbes and leads to drug resistance. The discovery of new and effective antibacterial agents or methods is quite a challenge because of its time and financially consuming nature. In addition, poor sanitation and increasing international travel have made transmission of infectious diseases easier.

This study focused on the development and optimizations of a sterilization technique using CAPJ utilizing the Taguchi method to shorten to experimental time spent on trial and error. In general, a design of orthogonal arrays of Taguchi analysis provides a maximum number of main factors to be estimated to minimize experimental trials. S/N ratios are transferred from the responses in the bactericidal activity by CAPJ treatment. ANOVA can further determine the contribution of CAPJ operating parameters on the preferable efficiency of antimicrobial condition. The effectiveness of Taguchi’s optimization approach is evaluated by antimicrobial effect, where 100% was obtained with the CAPJ using parameters obtained from the Taguchi method. As determined by the Taguchi method, the optimal antimicrobial activity is achieved with CAPJ parameters of application voltage 8.5 kV, APJ-sample distance 10 mm, Ar gas flow rate 500 sccm, and treatment time 300 s. We found CAPJ treatment time affected the plasma’s efficacy the most, and it required 5 min of treatment to achieve 100% bactericidal activity. By extension, the principles and concepts of Taguchi approach can also benefit industry reducing experimental trials of the performance, quality, and cost [22,33].

A number of studies represent very promising results for infection control by atmospheric pressure plasma [25]. In this work, we demonstrated that CAPJ built in our lab inhibited bacterial growth. The beneficial effects of plasma in sterilization are not yet fully understood, so we used our CAPJ to explore proposed mechanisms described in the literature. The observed temperatures of this CAPJ clearly indicated that consecutive plasma treatment by CAPJ remains below human body temperature for 30 min without causing thermal injury by utilizing the application voltage lower than 8.5 kV (Figure 2), which is an important requirement for biological applications. Previous studies reported that reactive oxygen, hydrogen peroxide, and UV photons produced by cold plasmas might target cell membrane and cell wall for antibacterial activity [34,35]. From the plasma diagnostics by OES (Figure 3), there is nearly no emission in the germicidal UV-C region around 254 nm [36]. The effect of heat and UV radiation as the main antimicrobial mechanism for plasma component, therefore, is unlikely.

On the other hand, The Ar mixed into He-based plasma described in this study contains OH and NO radicals, nitrogen and oxygen species, and metastable species of He and Ar (Figure 3), which probably interact with biological organisms to generate further reactive species [37]. Previous reports demonstrated ROS from different technologies might target different components of bacterial cells [38], which subsequently leads to the destruction of bacterial cell wall to achieve antimicrobial strategies. For example, ROS attacks the polyunsaturated fatty acids of the fatty acid membrane to initiate a self-propagating chain reaction [39], induces lipid peroxidation in Gram-negative bacteria [40], decomposes macromolecules such as DNA and protein [41], and breaks important C–O, C–N and C–C bonds of the peptidoglycan structure [42]. The distinguished emission at 309 nm in Figure 3 is a measurement for substantial amount of OH radicals, which are produced by plasma chemical reactions of dissociation and excitation of water molecules present in the air and are likely the part of plasma that is lethal toward living bacterial cells [43,44]. The intensity of OH produced by CAPJ is influenced by the feed gas mixture and increases with increasing Ar flow rate similar to a previous report [45]. The amount of OH radicals positively correlates to the antimicrobial efficiency as well. Deleterious OH radicals are the dominant reactive species and play a significant role in cell death [46,47].

It has been known that bacterial colonization of wounds exacerbated inflammation around the injury sites and slowed the skin healing. A previous study in bacteria infected skin diseases shows that *E. coli* is one of the four main Gram-negative bacteria among 90 isolated bacteria cultured from skin ulcers [48]. In the present study, we first assessed whether CAPJ possessed bactericidal effects on pathogenic *E. coli* and validated the bactericidal activity by using the animal experiment. We showed that S10 CAPJ treatment dramatically decreased bacterial load on day 4 as compared to untreated rat. However, we did not identify the bacterial species that were presented on the wounds. Our results are in line with previous in vitro studies, which showed that cold atmospheric plasma can decrease bacterial load independent of the strains [49,50]. Although CAPJ can effectively reduce bacterial loads in the animal study, further identification of bacterial species is worth studying in the future research.

**Figure 10:**
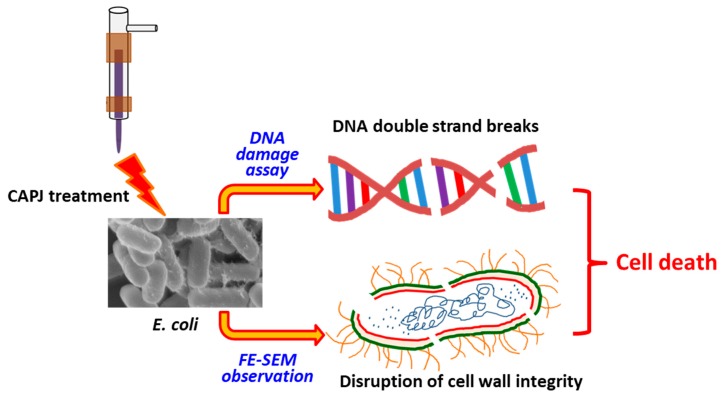
Antimicrobial mechanism of CAPJ treatment. The mechanism of the antimicrobial efficiency by CAPJ is suggested to kill the bacteria by destroying the cell wall of *E. coli*, damaging its DNA structure.

According to the observation of both the DNA damage assay (Figure 7) and the morphology of bacteria after CAPJ treatment by FE-SEM (Figure 8), CAPJ disrupts bacterial cell walls and induces DNA damage to exercise its antimicrobial effect in plasma mediated reactions. Figure 10 is a schematic that suggests the antimicrobial mechanism of CAPJ treatment noted in this study.

## 5. Conclusions

In this work, experimental design using the Taguchi method with a L9 orthogonal array was confirmed by S/N ratios and ANOVA, to optimize the operating parameters of CAPJ, achieving the best antimicrobial efficacy against *E. coli*. Parameters obtained via the Taguchi method were confirmed by 100% antimicrobial activity, with the final parameters of 8.5 kV CAPJ application voltage, 10 mm CAPJ-sample distance, 500 sccm Ar gas flow rate, and 300 s CAPJ treatment time. These parameters were further applied to wounds created on a rat model and showed a marked decrease in microbial load compared to an untreated wound, suggesting CPAJ have safe and effective application in vivo. As the intensity of hydroxyl radical produced by CAPJ is positively correlated to its antimicrobial efficiency, reactive species likely play a significant role for the plasma sterilization in this study. According to the observation of both the DNA damage assay and the bacterial cell wall integrity test after CAPJ treatment, the antimicrobial mechanism of CAPJ works through cell wall destruction and further DNA damage, thus ensuring antimicrobial activity. This makes CAPJ a promising and effective antimicrobial technique.

## Figures and Tables

**Table 1:** Factors and levels of CAPJ parameters for the antimicrobial of *E. coli.*

Symbol	Process Parameter	Level 1	Level 2	Level 3
A	Application voltage (kV)	6.5	7.5	8.5
B	CAPJ-sample distance (mm)	10	20	30
C	Ar gas flow rate (sccm)	0	200	500
D	CAPJ treatment time (s)	60	180	300

**Table 2:** Antimicrobial conditions of *E. coli* using the Taguchi L9 orthogonal array table. Symbols and numbers of control factors reflect the parameters and levels in Table 1.

Sample #	Control Factors			
	A	B	C	D
S1	1	1	1	1
S2	1	2	2	2
S3	1	3	3	3
S4	2	1	2	3
S5	2	2	3	1
S6	2	3	1	2
S7	3	1	3	2
S8	3	2	1	3
S9	3	3	2	1

**Table 3:** The Taguchi L9 sample designation and detailed CAPJ parameters.

Sample Designation		S1	S2	S3	S4	S5	S6	S7	S8	S9
**CAPJ conditions**	A (kV)	6.5	6.5	6.5	7.5	7.5	7.5	8.5	8.5	8.5
	B (mm)	10	20	30	10	20	30	10	20	30
	C (sccm)	0	200	500	200	500	0	500	0	200
	D (s)	60	180	300	300	60	180	180	300	60

**Table 4:** The bactericidal activity of *E. coli* by nine different CAPJ treatments.

Sample Designation.	S1	S2	S3	S4	S5	S6	S7	S8	S9
**Bactericidal activity (%)**	45.7	31.3	90.6	92.8	53.2	37.7	85.9	100.0	22.6

**Table 5:** Summary of the ANOVA results for the bactericidal activity of *E. coli* by CAPJ.

Source of Variance		Degree of Freedom	Sum of Square	Variance	Contribution (%)
A,	Application voltage (kV)	2	0.68	0.01	0.2
B,	CAPJ-sample distance (mm)	2	34.04	0.69	11.2
C,	Ar gas flow rate (sccm)	2	36.15	0.74	12.1
D,	CAPJ treatment time (s)	2	111.04	4.70	76.5
Total		8	181.91	6.15	100.0
